# A New Cardiac Channelopathy: From Clinical Phenotypes to Molecular Mechanisms Associated With Na_v_1.5 Gating Pores

**DOI:** 10.3389/fcvm.2018.00139

**Published:** 2018-10-09

**Authors:** Adrien Moreau, Mohamed Chahine

**Affiliations:** ^1^PhyMedExp, Université de Montpellier, INSERM, CNRS, Montpellier, France; ^2^CERVO Research Centre, Institut Universitaire en Santé Mentale de Québec, Quebec City, QC, Canada; ^3^Department of Medicine, Université Laval, Quebec City, QC, Canada

**Keywords:** gating pore current, SCN5A, Na_v_1.5, dilated cardiomyopathy, cardiomyocytes, cardiac arrhythmias

## Abstract

Voltage gated sodium channels (Na_V_) are broadly expressed in the human body. They are responsible for the initiation of action potentials in excitable cells. They also underlie several physiological processes such as cognitive, sensitive, motor, and cardiac functions. The Na_V_1.5 channel is the main Na_V_ expressed in the heart. A dysfunction of this channel is usually associated with the development of pure electrical disorders such as long QT syndrome, Brugada syndrome, sinus node dysfunction, atrial fibrillation, and cardiac conduction disorders. However, mutations of Na_v_1.5 have recently been linked to the development of an atypical clinical entity combining complex arrhythmias and dilated cardiomyopathy. Although several Na_v_1.5 mutations have been linked to dilated cardiomyopathy phenotypes, their pathogenic mechanisms remain to be elucidated. The gating pore may constitute a common biophysical defect for all Na_V_1.5 mutations located in the channel's VSDs. The creation of such a gating pore may disrupt the ionic homeostasis of cardiomyocytes, affecting electrical signals, cell morphology, and cardiac myocyte function. The main objective of this article is to review the concept of gating pores and their role in structural heart diseases and to discuss potential pharmacological treatments.

## Introduction

Cardiovascular diseases are the single most common cause of death worldwide, and sudden deaths due to cardiac arrhythmias account for ~50% of these deaths (1). Heart failure (HF) is a major public health problem in industrialized countries, in particular because of its frequency and its consequences in terms of morbidity and mortality (2). The financial costs of heart failure (HF) are substantial and are increasing constantly due to higher healthcare costs, improved therapies that extend life expectancy, and an aging population.

Dilated cardiomyopathy (DCM) is the most common cause of HF in North America. It induces the dilatation of cardiac cavities and impairs contractility and systolic function (3). It accounts for over 90% of all cardiomyopathy cases referred to specialized centers and is collectively the most common reason for heart transplants in the young (4, 5). Familial or genetically related DCM make up 20 to 30% of DCM cases. Most genes associated with DCM encode structural proteins involved in contractile function and the cytoskeletal matrix. Mutations in genes encoding these proteins are believed to diminish the overall structural integrity of cells, leading to myocyte disarray, the development of fibrosis characteristic of DCM, and myocyte death (3, 6). Sodium (SCN5A), and potassium (ABCC9, K_ATP_) channel regulation defects have also been associated with the development of DCM, which argues for an alternative disease mechanism of dilatation-induced remodeling that is mainly driven by a dysfunction in an electrical excitability component rather than a primary structural defect (6, 7).

The development of effective drugs has markedly improved the prognosis of patients with HF. Four therapeutic classes have demonstrated efficacy in the management of HF. These include angiotensin-converting enzyme inhibitors such as enalapril, Angiotensin Receptor blockers such as losartan, aldosterone inhibitors such as spironolactone, and beta-blockers such as carvedilol, metoprolol, and bisoprolol (8). A combination of different pharmacological treatments may help limit the pathological remodeling responsible for the evolution of the disease. In addition to the long-term treatment of HF, diuretics are prescribed to limit the appearance of edema (9).

The purpose of this review is to explore the mechanisms involved in *SCN5A* mutations linked to DCM, with a focus on their role in generating gating pore currents as a potentially unifying molecular mechanism.

## Voltage-gated Na^+^ channels

Voltage-gated Na^+^ channels are transmembrane proteins that play a critical role in action potential (AP) initiation and propagation in many excitable cells and thus constitute the driving force for generating electrical impulses. The dysfunction of voltage-gated Na^+^ channels has been reported to affect activity in skeletal muscle, the heart, and the nervous system, causing a variety of diseases such as paralysis, cardiac arrhythmic disorders such as disturbances in cardiac conduction (10), type 3 long QT syndrome (11), Brugada syndrome (BrS) (12), cardiac conduction defect (CCD) (13), pain, and epilepsy (14). Na^+^ channels are composed one α-subunit (260 kDa) associated with one or more accessory β-subunits (β_1_-β_4_) (15). Channel function and kinetics are primarily driven by pore-forming α-subunits and are modulated by β-subunits. All Na^+^ channel α-subunits comprise four homologous domains (DI-DIV), each of which contains six transmembrane segments (S1-S6). S1-S4 form the voltage sensor domain and S5-S6 form the pore domain, with a hairpin-like P-loop located between S5 and S6 (16, 17). The short linkers connecting S5 and S6 form the outer narrow mouth of the pore and the selectivity filter, while the inner wider pore is formed by the S5 and S6 segments. The S4 segments in each voltage sensor domain contain positively charged amino acid residues that act as gating charges and move across the membrane to trigger channel activation in response to membrane depolarization (18). The short intracellular cytoplasmic loop connecting homologous domains III and IV acts as the inactivation gate, which bends back into the channel and blocks the pore from the intracellular side during sustained depolarization of the membrane. The inactivation gate is located in the center of a three-amino-acid stretch consisting of isoleucine, phenylalanine, and methionine (IFM) (19). Residues of the S6 segments in each domain provide the binding site for local anesthetics and link the internal vestibule (20). The α-subunit is the major component of the channel. In a heterologous expression system, it recapitulates all the wilde type channel's main biophysical properties (16).

## Cardiac muscle Na^+^ channel legacy

The *SCN5A* gene encodes the cardiac Na^+^ channel known as Na_V_1.5, a member of an evolutionarily highly conserved family of voltage-gated ion channels. The *SCN5A* gene is located on chromosome 3p21 and was initially called hH1 for human heart Na^+^ channel 1 (16). Na_v_1.5 is the main Na^+^ channel expressed in the heart. It is also present at high levels in the piriform cortex (larger part of the olfactory system) and subcortical limbic nuclei (21). Na_V_1.5 is much more TTX-resistant than skeletal muscle or central nervous system sodium channels, requiring much higher concentrations of TTX (micromolar concentrations) to be inhibited. This relative resistance is due to the presence of certain amino acid residues, in particular a cysteine instead of an aromatic residue in the P-region of DI (22, 23). On the other hand, Na_v_1.5 is more sensitive to inhibition by local anesthetics such as lidocaine and antiarrhythmic agents than peripheral nervous system (PNS) channels and has a more negative voltage-dependence of inactivation than PNS channels (16, 24). Mutations in *SCN5A* have been primarily associated with pure arrhythmic disorders such as long QT syndromes (LQTs), Brugada syndrome (BrS), atrial fibrillation (AFib), progressive cardiac conduction defect (PCCD), and sinus node dysfuction (SND), all of which are inherited cardiac diseases. The most common phenotypes caused by mutations in *SCN5A* are LQTS type 3 (LQT3) (25) and BrS (26). Both syndromes are diagnosed by irregularities on surface ECGs, with no apparent structural heart abnormalities, and can lead to malignant ventricular arrhythmias or even sudden cardiac death (SCD). The different clinical and ECG phenotypes of LQT3 and BrS arise from opposing specific alterations in the biophysical mechanisms associated with cardiac Na^+^ channel dysfunction. LQT3 is caused by *SCN5A* mutations that result in a gain of channel function, a disruption in fast inactivation, and the appearance of a persistent Na^+^ current. A gain of function consists of a higher quantity of Na^+^ flowing through the channel during a stimulation. In contrast, BrS is caused by a loss of channel function, and thus a lower amount of Na^+^ flowing through the channel during a stimulation (27, 28). In addition to BrS and LQT3, *SCN5A* variants have also been associated with PCCD. Like BrS, PCCD variants result in a loss of function of Na^+^ channels. PCCD and BrS loss-of-function phenotypes are closely related as shown by the fact that three of the six known PCCD variants are also associated with BrS. Only a few *SCN5A* mutations are known to cause such mixed phenotypes, which are purely electrical in nature, with no structural abnormalities (29, 30). Bezzina et al. described the first *SCN5A* mutation (1795insD) that caused both BrS and LQTS in the same affected individuals of a large family (29). The biophysical characterization revealed balanced defects, with mutated channels displaying both gain and loss of function.

However, mutations in *SCN5A* do not just lead to pure arrhythmic disorders. They can also be associated with structural heart diseases. Distinct cardiac phenotypes caused by *SCN5A* mutations have also been described, including SND and conduction disorder associated with DCM. It is not well understood how a dysfunction in electrical excitability through altered Na^+^ channel function may underlie the manifestation of dilatation remodeling and DCM.

**Figure 1:**
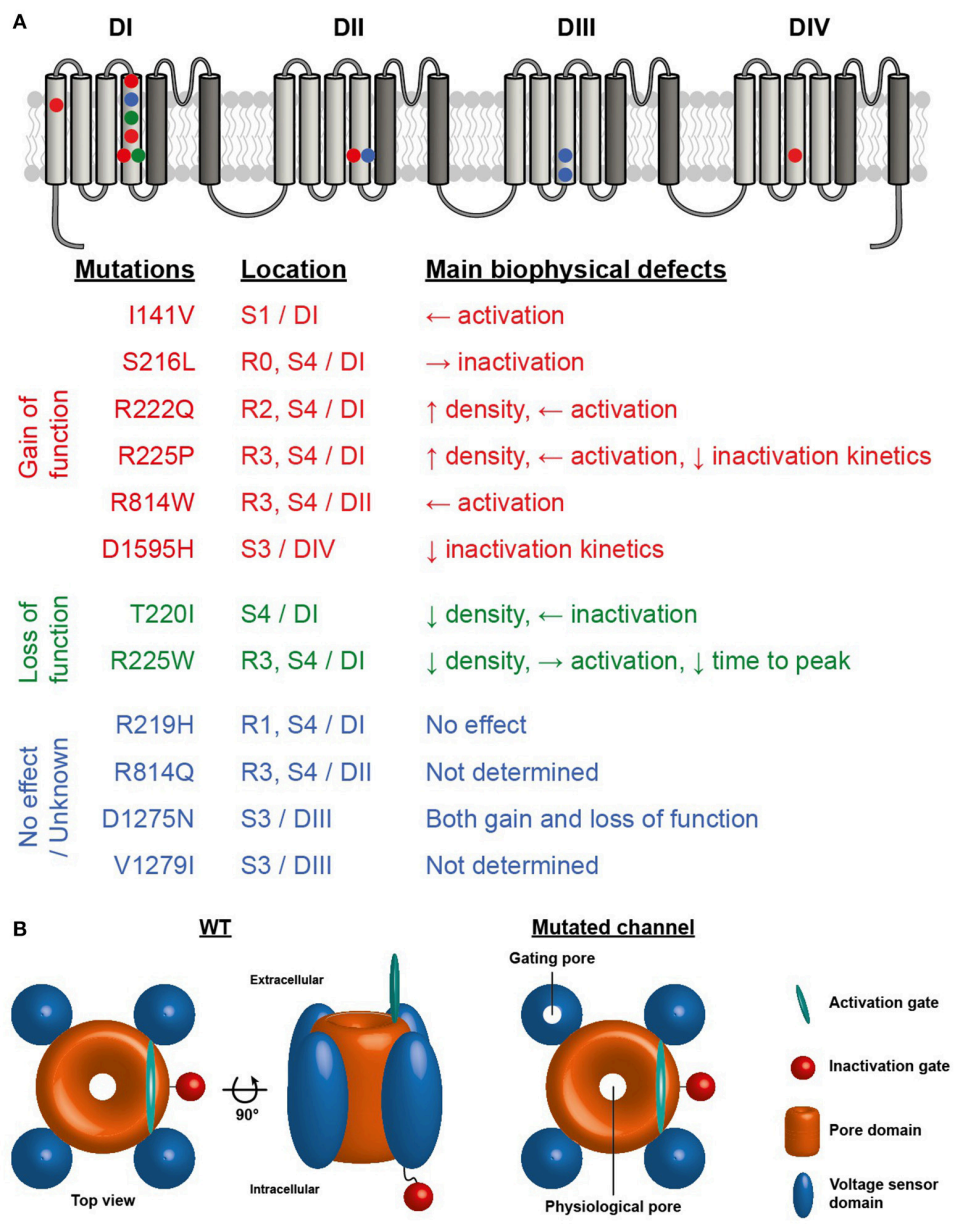
**(A)** Structure of Na_V_1.5 voltage-gated sodium channel illustrating the four homologous domains of the channel. The VSD formed by the S1-S4 segments of each domain are represented by light gray segments. The dark gray segments represent the pore domain of the channel. Symbols indicate the locations of the Na_V_1.5 VSD mutations associated with the development of cardiac arrhythmias and DCM. Red symbols mutation reported to cause gain of function, green symbols loss of function and blue symbols no effect or no known effect on the channel. **(B)** Schematic representation of Na_V_1.5 sodium channel illustrating the central pore and the presence of a gating pore in one of the VSDs.

The first report linking Na^+^ channel dysregulation to the etiology of DCM was published in 2004 by McNair and coworkers, while the same mutation was previously published in 2003 but without any cardiac dilatation phenotype (7, 31). A missense mutation in *SCN5A* (D1275N) was associated with a dilatation phenotype in a pedigree characterized by cardiac arrhythmias and sudden death (7). Echocardiographic data indicated cardiac dilatation in the carriers. Of note, among the 8 affected family members, 3 also demonstrated allelic variations in the promoter region and first exon of the *Cx40 gene*. In 2003, the electrophysiological characterization of the mutant Na^+^ channels using the *Xenopus* oocyte expression system revealed enhanced channel activation (31). In 2005, letters from both teams further hypothesized that the dilation observed could also have been caused by a combination of modifier genes, or environmental or unknown factors acting in conjunction with the primary Na^+^ ion conduction defect (32). In a more recent study of a cohort of 338 DCM patients, McNair et al. estimated that a dysfunction of Na_v_1.5 proteins causes 1.7% of familial DCM cases (33). Indeed, the *SCN5A* gene is ranked as the sixth most common cause of familial DCM (3). To date, 12 *SCN5A* mutations have been linked to complex arrhythmia disorders and DCM, including the R219H mutation recently reported by our group (34). Interestingly, nine of these mutations involve highly conserved residues on the VSD, mainly on the S3 and S4 transmembrane segments, which play a pivotal role in channel activation (33). VSD mutations have been implicated in generating leak currents known as gating pore currents or omega currents in neuromuscular disorders (35). Intriguingly, it has recently been shown that *SCN5A* mutations in patients with DCM combined with complex arrhythmias have either gain and/or loss of function biophysical phenotypes when explored in a heterologous expression system (36) (see Figure 1 for a summary of the locations and biophysical properties of these mutants). However, at this juncture, it is unclear which mechanism is involved in the *SCN5A*-linked pathogenesis of DCM. Gating pore currents are cation currents that selectively flow through the mutated VSDs of Na^+^ channels and their biophysical properties are directly related to the movement of the voltage sensor. These currents do not reflect pore activity since pore blockers such as tetrodotoxin (TTX) do not affect them. Similar H^+^ channels can be formed by replacing the most positively charged arginine residue of the *Drosophila Shaker* voltage-gated K^+^ channel with a histidine (37). Our central hypothesis is that mutation-induced gating pore currents through the Na_v_1.5 VSD may underlie the biophysical phenotype in DCM.

To better understand the complex relationship of *SCN5A*-linked DCM mutations, Watanabe et al. created a humanized mouse model that harbors the D1275N *SCN5A* mutation. They concluded that the D1275N variant is a pathological mutation that causes conduction slowing, arrhythmias, and DCM phenotypes (38). However, this is not representative of *SCN5A*-linked DCM mutations that are present on different Na_V_1.5 domains, and it fails to explain the molecular mechanisms underlying DCM phenotypes.

## What is a gating pore?

The very first instance of ions flowing directly through the VSD of a voltage sensitive ion channel was reported in 2004 by Starace and Bezanilla (37). At the time, the authors were focused on describing the structure and function of VSDs (39). They showed that the substitution of the first arginine (R1) of the S4 segment (R1/S4) by a histidine led to an aberrant H^+^-specific current (37). As this H^+^ flow was not sensitive to the block of the physiological pore of the protein, the authors concluded that ions did not pass through this structure but rather directly through the VSD (37). This concept was rapidly extended to the substitution of R1/S4 by other amino acids such as alanine (A), cysteine (C), serine (S), and valine (V) (40). The newly created current was not specific to H^+^ but was cation selective. The location of the permeation pathway was further refined using the combination of the R1C mutation and MTSET. The gating pore current was blocked by the addition of MTSET, which forms disulfide bonds with cysteines, thus confirming the location of the permeation pathway (40). At this point, the newly created permeation pathway was called an omega pore in opposition to the physiological alpha pore of the channel protein. The initial arginines of the S4 segment were thought to naturally obstruct this unusual permeation pathway. Their substitution with “smaller” amino acids would thus leave a gap, allowing the permeation of ions. All these experiments were performed using the *Drosophila Shaker* voltage-gated K^+^ channel. In contrast, a gating pore current in a mammalian voltage gated Na^+^ channel (Na_v_1.2) was first observed in the setting of a double S4-R1G/R2G substitution in DII (41). Besides the double substitution, the resulting current was of small amplitude. Indeed, due to the monomeric nature of Na_v_ channels, each channel presents only a single gating pore. On the other hand, the tetrameric nature of K_v_ channels leads to four identical gating pores for each functional channel.

The biophysical properties of gating pores influence the ion flow. Most of the gating pores that have been described are created by the substitution of S4 arginines. Due to their location, the biophysical properties of gating pores are intimately linked to the function of VSDs. As previously mentioned, VSDs are made up of four transmembrane segments (S1-S4) containing two structures: the positively charged S4 segment and the surrounding stabilizing S1-S3 segments (16, 17, 42, 43). Stabilization is notably ensured by the gating charge transfer center (GCTC), a specific arrangement of two negatively charged residues on S2 and S3 and an aromatic amino acid on S2 (43). In voltage gated ion channels, the VSD is the structure responsible for sensing changes in membrane potential. During a depolarization, the S4 segment undergoes a large outward movement in which each S4 arginine sequentially interacts with the GCTC (44–46). This charge movement can be monitored as a function of membrane voltage and gives the Q-V curve, which describes the two main stable states of the VSD: the resting and the activated states. In the resting state (hyperpolarized voltages), the S4 uppermost arginines interact with the GCTC while, in the activated state (depolarized voltages), the S4 innermost arginines preferentially interact with the GCTC (44–46). In WT VSDs, tight interactions between the S4 arginines and the GCTC create hydrophobic septa that isolate water crevices on both sides of the membrane, ensuring a non-permeable VSD (47–49). Gating pores are created by the disruption of interactions between the S4 and the GCTC, leading to the junction of the water crevices (50–52). The recently reported crystal structure of WT and mutated bacterial Na_v_Ab channels have provided support for this experimental and molecular dynamic simulation-based hypothesis (49). In their study, after measuring the gating pores created by mutating the second and third S4 arginines, Jiang et al. created and studied the corresponding crystal structures (49). Their study thus provides a strong basis for explaining the atomic mechanism underlying the creation of gating pores. As such, mutations affecting the uppermost S4 arginines disrupt interactions when the VSD is in its resting state, and the permeation pathway allows ions to flow at hyperpolarized voltages (34, 49, 50). On the other hand, mutations affecting the innermost S4 arginines disrupt interactions in the activated state, leading to gating pore currents at depolarized voltages (49, 52, 53). Opening probabilities for gating pores thus depend on the Q-V of the mutated VSD.

Gating pores are permeation pathways located directly inside VSDs, a usually non-conductive structure. Consequently, unlike physiological alpha pores, gating pores do not benefit from dedicated specific selectivity filters. However, two main sub-types of gating pores can be distinguished: (i) cation-selective and (ii) H^+^-specific gating pores (34, 35, 37, 39–41, 50, 54–61). Cation-selective gating pores are created by the substitution of S4 arginines for amino acids other than histidine. In this setting, based on published selectivity sequences, large cations (below the exclusion size) preferentially flow through the gating pore (35, 40, 52, 60–62). Anions are excluded because of the lack of a positive charge due to the arginine substitution (63, 64). H^+^-specific gating pore currents can be considered as special cases as they are related to the substitution of S4 arginines by histidines. Histidine is the only natural amino acid with a pKa of 6.5. Consequently, at physiological pH, histidine can link and release H^+^. Interestingly, in two independent studies, half of the maximal measured gating pore current was observed at pH 6.48 and 6.5, values very close to the pKa of histidine (34, 37). In the specific case of R-to-H substitutions, H^+^ permeation occurs through a “*Grotthus hopping*” mechanism where a H^+^ is linked to histidine while another is released at the opposite side (37).

Lastly, gating pores can display different voltage dependence and ion selectivity depending on the nature of the mutation and its location in the VSD. S1 and S3 mutations such as I141V (S1/DI), D1275N (S3/DIII), V1279I (S3/DIII), and D1595H (S3/DIV) would be expected to open a permeation pathway since creating a gating pore relies on the disruption of interactions between S4 and the GCTC. However, very few gating pores generated by mutations outside the S4 segment have been described (54, 65). Further work on S1-S3 mutations is clearly required to better understand the biophysical properties of potentially novel permeation pathways.

## Downstream consequences of gating pores

The cardiac consequences of gating pores remain a matter of debate given that no specific studies have explored this issue to date. Cardiac defects potentially caused by an omega current remain hypothetical. Such studies would require a mutation that does not affect the alpha pore properties of the channel in order to properly isolate defects linked solely to the gating pore. Due to the low amplitude of gating pore currents, their pathological nature is often questioned. However, two major observations argue in favor of gating pores being truly deleterious: (i) gating pores in Na_v_1.4 and Ca_v_1.1 are commonly accepted as the cause of hypokalemic/normokalemic periodic paralysis (35, 55, 58, 59, 66–68), and (ii) despite an amplitude that is comparable to omega currents, persistent currents related to Na_v_1.5 LQT3 mutations are also commonly recognized as the original cause of LQT3 syndrome (69, 70). In fact, the small amplitude of gating pore currents appears to be compensated by the time during which the aberrant permeation pathway is in a conductive state. Na_v_1.5/S216L, R219H, and T220I mutations affect the outermost S4 residues and have been associated with the development of arrhythmias and DCM (34, 51, 71, 72). Based on their location (Na_v_1.5/S216L, T220I) and on experimental results (Na_v_1.5/ R219H), these mutations open (or are expected to open) a gating pore at hyperpolarized voltages. Na^+^ or H^+^ ions (depending on the mutation) would thus flow during diastole as soon as the VSDs are in their resting state (34, 50). On the other hand, Na_v_1.5 S4 mutations associated with DCM such as Na_v_1.5/R222Q, R225W, R225P, R225Q, R814Q, and R814W affect the intermediate or innermost S4 residues (52, 53). The resulting gating pore is mainly opened (or expected to be open) under depolarized conditions (52, 53). However, the biophysical properties of gating pores that open at depolarized voltages appear slightly more complex. Indeed, ions flow as soon as the VSDs are in their activated state (during systole) but, due to the relaxation process of the S4 segment after prolonged depolarizations (several hundred of milliseconds), the gating pores remain temporarily conductive at hyperpolarized voltages. This leads to a major K^+^ outflow at depolarized potentials and a transient Na^+^ inflow at hyperpolarized voltages (52, 53, 62).

Gating pores in all configurations are thought to induce a global Na^+^ overload. This process has been observed directly in patients suffering from HypoPP by Na^+^ magnetic resonance imaging (67). In the case of H^+^-specific gating pore currents, the Na^+^ overload relies on the Na^+^/H^+^ exchanger working to attenuate the intracellular acidification caused by the increased H^+^ concentration. The Na^+^ overload is thought to lead to a Ca^2+^ overload by way of the Na^+^/Ca^2+^ exchanger, suggesting that it has a major impact on cellular ionic homeostasis (73). Such Ca^2+^ overload could also be pro-arrhythmogenic by it-self. This ionic unbalance is known to block inward rectifier potassium channels (K_ir_ channels). K_ir_ have been described to play a major role in setting the resting membrane potential (V_Rest_) and also affect the duration of APs (74, 75). While the effect of their blockade should be further studied, it could depolarize the V_Rest_ and potentially lengthen APs, establishing a highly pro-arrhythmogenic substrate. Furthermore, despite its limited effect, Ca^2+^ has also been described as modulating the rectification of I_K_ channels thus potentially participating to the AP lengthening (76). K_ir_ blockade most probably resulting in depolarized V_Rest_ has also been described in the pathogenic process of HypoPP and has been attributed to a gating pore current (58, 67, 77, 78). Both cellular acidification and a Ca^2+^ overload impair connexin coupling and thus cell-cell conduction (79–81), further decreasing the conduction velocity. Furthermore, most of Na_v_1.5 mutations linked to DCM also demonstrate primary biophysical defects (gain or loss of function). Taken together, these primary biophysical defects and gating pores most probably explain the conduction disorders that are often observed in patients carrying Na_v_1.5 mutations and suffering from complex arrhythmias associated with DCM (33, 34, 82–84). Cellular acidification is strongly suspected in the case of H^+^-specific gating pore currents. Based on studies performed in different contexts, cytoplasm acidification has been reported to lengthen APs (85). Interestingly, the opposite process (increase in intracellular pH) has recently been reported to induce AP shortening (86). The proposed cardiac consequences likely constitute a highly pro-arrhythmic substrate most probably participating in the development of electrical dysfunctions reported in affected patients. Interestingly, to get further insights in cardiac electrical effect of a gating pore *in-silico* modeling experiments could reveal to be highly valuable. However, so far, despite the availability of several models of cardiac cellular electrophysiology, further studies are required to develop such insightful adaptations.

In addition to electrical dysfunctions, ionic homeostasis imbalances have been reported to dramatically affect structural protein function. For example, intracellular acidosis decreases the affinity of troponin C for calcium, resulting in excitation-contraction impairment (87). Furthermore, a Ca^2+^ overload leads to partial cardiomyocyte decrease in force contraction and impaired myofilament function (88, 89). Taken together, the consequences of ionic homeostasis imbalances weaken the heart structure against a background of unchanging blood pressure, progressively leading to heart chamber dilatation. It was initially proposed that dilatation in patients carrying Na_v_1.5 mutations might rely on deleterious adaptive heart remodeling. However, the report of the Na_v_1.5/R225W mutation in a 1-year-old child who died from severe arrhythmias and DCM ruled out potential remodeling in this patient (83). In a nutshell, gating pore currents are expected to affect V_Rest_, AP parameters, cellular conduction, and cardiomyocyte structure, all of which might act together to cause multiple arrhythmias associated with cardiac dilatation.

As previously mentioned, Na_v_1.5 mutations in the VSD that are outside the S4 segment such as Na_v_1.5/I141V, D1275N, V1279I, and D1595H should be treated with caution. D1275N was the first Na_v_1.5 mutation associated with the development of arrhythmias and DCM (7). It is also one of the most studied Na_v_1.5 DCM-linked mutations (7, 38, 71, 90–93). Besides the marked interest in this mutation and the many ensuing experimental models, the molecular mechanism linking the Na_v_1.5/D1275N mutation and its pathological expression remains unclear. The clinical phenotype is variable, and heart defects include atrial and ventricular arrhythmias and conduction system defects and, most of the time, cardiac dilatation (7, 38, 71, 90–93). Strikingly, Na_v_1.5/D1275N is also one of the only Na_v_1.5 mutations that have been linked to cerebro-vascular strokes (7, 90, 93, 94). In heterologous expression systems, only mild defects, mostly shifts in activation/inactivation parameters and current decreases, have been described (91). Further investigations using a humanized mouse model indicated that there is a large reduction in current amplitude (38). The lengthened cardiac conduction time (lengthened PR interval) in transgenic Na_v_1.5/D1275N zebrafish appear to support this hypothesis. However, in this case, the Na_v_ current was not measured (95). Lastly, a human cardiac cellular model of the cardiomyopathy linked to the Na_v_1.5/D1275N mutation has been recently proposed based on patient-specific induced pluripotent stem cells (hiPSC) (93). In this study, the authors only report a decrease in the Na^+^ current amplitude and a mild shift of activation leading to a decrease in the maximal depolarization velocity of APs (93). Unfortunately, they did not study intracellular ionic homeostasis and sarcomeric arrangements. Taken together, these studies identified a biophysical defect that does not provide a complete picture of the clinical expression of this mutation. Interestingly, given the location of the D1275N mutation (S3/DIII), a gating pore could be created and participate in the pathological mechanism. However, this hypothesis has never been extensively explored. Furthermore, as previously mentioned, due to the structure and function of the S1–S3 segments, gating pores created by mutations in these segments are not expected to behave in the same way as gating pores created by mutations in the S4 segment. Characterizing and understanding them will thus require thorough studies.

Finally, a specific gating pore blocker would be expected to be ideal to efficiently treat gating pore pathological consequences. Unfortunately, so far, such universal blocker has never been described. Consequently, the only option available is to alleviate the cardiac dilatation and use anti-arrhythmic or implantable devices to slow down the pathology progress. In a research optic, few studies already proposed potential blockers such as divalent (Mg^2+^), trivalent (Y^3+^, Yb^3+^, Lu^3+^, Ti^3+^) and quadrivalent (Hf^4+^) cations (40, 59). In WT channels, gating pores are naturally obstructed by the arginine side, notably made of guanidine (49, 59). Guanidinium compounds have recently been shown to bind to the VSD in the place of the missing side chain of the original arginine (49). Still supporting this hypothesis, the 1-(2,4-xylyl)guanidine, a guanidinium derivative has been shown to partially block gating pores (59). While obstructing gating pores is an ongoing challenge, other approaches could be valuable. Since gating pores properties intimately depends on the VSD characteristics, VSD modulation through the use of toxins has been demonstrated to modulate the voltage dependence of gating pores (96).

In this review, we describe the history of Na_v_1.5 associated channelopathies with a clear focus on the history of Na_v_1.5 mutations more recently associated with the development of multiple arrhythmias and DCM. The biophysical function of the VSDs, the creation of a gating pore and their biophysical properties have been described. Finally, the potential cardiac cellular effects of gating pores and their blockers are also presented here. The increasing knowledge regarding gating pores and their pathologic implication, potentially highlights novel biophysical defects and consequently novel channelopathies. In the current dynamic toward more precise and personalized medicine, this increasing knowledge could in the future orientate clinicians in their day to day practice in the management of cardiac channelopathies. This could also help to develop specifically targeted novel medication to accurately and precisely block gating pores, finally benefitting patients. The increased interest in gating pores highlights VSDs as highly valuable druggable sites with potential impact far beyond field of gating pore currents. So far, most ions channel modulators only target the physiological pore of the channel. Modulating the VSD would thus offer a wide range of benefits and be an approach to consider in most channelopathies.

## Author contributions

All authors listed have made a substantial, direct and intellectual contribution to the work, and approved it for publication.

### Conflict of interest statement

The authors declare that the research was conducted in the absence of any commercial or financial relationships that could be construed as a potential conflict of interest.

## References

[1] Estes NA III. Predicting and preventing sudden cardiac death. Circulation (2011) 124:651–6. 10.1161/CIRCULATIONAHA.110.974170 21810674

[2] Savarese G Lund LH. Global public health burden of heart failure. Card Fail Rev. (2017) 3:7–11. 10.15420/cfr.2016:25:2 28785469 PMC5494150

[3] Hershberger RE Siegfried JD. Update 2011: clinical and genetic issues in familial dilated cardiomyopathy. J Am Coll Cardiol. (2011) 57:1641–9. 10.1016/j.jacc.2011.01.015 21492761 PMC3088091

[4] Murphy RT Starling RC. Genetics and cardiomyopathy: where are we now? Cleve Clin J Med. (2005) 72:465–466, 469–70, 472–63 passim. 16018289 10.3949/ccjm.72.6.465

[5] Taylor MR Carniel E Mestroni L. Cardiomyopathy, familial dilated. Orphanet J Rare Dis. (2006) 1:27. 10.1186/1750-1172-1-27 16839424 PMC1559590

[6] Bienengraeber M Olson TM Selivanov VA Kathmann EC O'cochlain F Gao F. ABCC9 mutations identified in human dilated cardiomyopathy disrupt catalytic KATP channel gating. Nat Genet. (2004) 36:382–7. 10.1038/ng1329 15034580 PMC1995438

[7] Mcnair WP Ku L Taylor MR Fain PR Dao D Wolfel E. SCN5A mutation associated with dilated cardiomyopathy, conduction disorder, and arrhythmia. Circulation (2004) 110:2163–7. 10.1161/01.CIR.0000144458.58660.BB 15466643

[8] Parikh R Kadowitz PJ. A review of current therapies used in the treatment of congestive heart failure. Exp Rev Cardiovasc Ther. (2013) 11:1171–8. 10.1586/14779072.2013.816478 23980607

[9] Habal MV Garan AR. Long-term management of end-stage heart failure. Best Pract Res Clin Anaesthesiol. (2017) 31:153–66. 10.1016/j.bpa.2017.07.003 29110789 PMC5726453

[10] Cummins TR Dib-Hajj SD Waxman SG. Electrophysiological properties of mutant Nav1.7 sodium channels in a painful inherited neuropathy. J.Neurosci. (2004) 24:8232–6. 10.1523/JNEUROSCI.2695-04.2004 15385606 PMC6729696

[11] Bennett PB Yazawa K Makita N George AL Jr. Molecular mechanism for an inherited cardiac arrhythmia. Nature (1995) 376:683–5. 10.1038/376683a0 7651517

[12] Brugada P Brugada J. Right bundle branch block, persistent ST segment elevation and sudden cardiac death: a distinct clinical and electrocardiographic syndrome. J Am Coll Cardiol. (1992) 20:1391–6. 10.1016/0735-1097(92)90253-J 1309182

[13] Veldkamp MW Viswanathan PC Bezzina C Baartscheer A Wilde AM Balser JR. Two distinct congenital arrhythmias evoked by a multidysfunctional Na^+^ channel. Circ.Res. (2000) 86:E91–7. 10.1161/01.RES.86.9.e91 10807877

[14] Meisler MH Kearney JA. Sodium channel mutations in epilepsy and other neurological disorders. J Clin Invest. (2005) 115:2010–7. 10.1172/JCI25466 16075041 PMC1180547

[15] Catterall WA. From ionic currents to molecular mechanisms: the structure and function of voltage-gated sodium channels. Neuron (2000) 26:13–25. 10.1016/S0896-6273(00)81133-2 10798388

[16] Gellens ME George AL Jr Chen LQ Chahine M Horn R Barchi RL. Primary structure and functional expression of the human cardiac tetrodotoxin-insensitive voltage-dependent sodium channel. Proc Natl Acad Sci USA. (1992) 89:554–8. 10.1073/pnas.89.2.554 1309946 PMC48277

[17] Terlau H Stuhmer W. Structure and function of voltage-gated ion channels. Naturwissenschaften (1998) 85:437–44. 10.1007/s001140050527 9802045

[18] Stühmer W Conti F Suzuki H Wang XD Noda M Yahagi N. Structural parts involved in activation and inactivation of the sodium channel. Nature (1989) 339:597–603. 10.1038/339597a0 2543931

[19] West JW Patton DE Scheuer T Wang Y Goldin AL Catterall WA. A cluster of hydrophobic amino acid residues required for fast Na^+^-channel inactivation. Proc Natl Acad Sci USA. (1992) 89:10910–4. 10.1073/pnas.89.22.10910 1332060 PMC50452

[20] Ragsdale DS Mcphee JC Scheuer T Catterall WA. Molecular determinants of state-dependent block of Na^+^ channels by local anesthetics. Science (1994) 265:1724–8. 10.1126/science.8085162 8085162

[21] Hartmann HA Colom LV Sutherland ML Noebels JL. Selective localization of cardiac SCN5A sodium channels in limbic regions of rat brain. Nat Neurosci. (1999) 2:593–5. 10.1038/10147 10404176

[22] Backx PH Yue DT Lawrence JH Marban E Tomaselli GF. Molecular localization of an ion-binding site within the pore of mammalian sodium channels. Science (1992) 257:248–51. 10.1126/science.1321496 1321496

[23] Heinemann SH Terlau H Imoto K. Molecular basis for pharmacological differences between brain and cardiac sodium channels. Pflügers Arch. (1992) 422:90–2. 10.1007/BF00381519 1331981

[24] Bean BP Cohen CJ Tsien RW. Lidocaine block of cardiac sodium channels. J Gen Physiol. (1983) 81:613–42. 10.1085/jgp.81.5.613 6306139 PMC2216565

[25] Splawski I Shen J Timothy KW Lehmann MH Priori S Robinson JL. Spectrum of mutations in long-QT syndrome genes. KVLQT1, HERG, SCN5A, KCNE1, and KCNE2. Circulation (2000) 102:1178–85. 10.1161/01.CIR.102.10.1178 10973849

[26] Chen Q Kirsch GE Zhang D Brugada R Brugada J Brugada P. Genetic basis and molecular mechanism for idiopathic ventricular fibrillation. Nature (1998) 392:293–6. 10.1038/32675 9521325

[27] Wang Q Shen J Splawski I Atkinson D Li Z Robinson JL. SCN5A mutations associated with an inherited cardiac arrhythmia, long QT syndrome. Cell (1995) 80:805–11. 10.1016/0092-8674(95)90359-3 7889574

[28] Herbert E Chahine M. Clinical aspects and physiopathology of Brugada syndrome: review of current concepts. Can J Physiol Pharmacol. (2006) 84:795–802. 10.1139/y06-038 17111025

[29] Bezzina C Veldkamp MW Van Den Berg MP Postma AV Rook MB Viersma JW. A single Na^+^ channel mutation causing both long-QT and Brugada syndromes. Circ Res. (1999) 85:1206–13. 10.1161/01.RES.85.12.1206 10590249

[30] Ter Bekke RMA Isaacs A Barysenka A Hoos MB Jongbloed JDH Hoorntje JCA. Heritability in a SCN5A-mutation founder population with increased female susceptibility to non-nocturnal ventricular tachyarrhythmia and sudden cardiac death. Heart Rhythm (2017) 14:1873–81. 10.1016/j.hrthm.2017.07.036 28782696

[31] Groenewegen WA Firouzi M Bezzina CR Vliex S Van Langen IM Sandkuijl L. A cardiac sodium channel mutation cosegregates with a rare connexin40 genotype in familial atrial standstill. Circ Res. (2003) 92:14–22. 10.1161/01.RES.0000050585.07097.D7 12522116

[32] Groenewegen WA Wilde AA Letter regarding article by McNair et al, “SCN5A mutation associated with dilated cardiomyopathy, conduction disorder, and arrhythmia”. Circulation (2005) 112:e9 10.1161/CIRCULATIONAHA.104.531475 15998690

[33] Mcnair WP Sinagra G Taylor MR Di Lenarda A Ferguson DA Salcedo EE. SCN5A mutations associate with arrhythmic dilated cardiomyopathy and commonly localize to the voltage-sensing mechanism. J Am Coll Cardiol. (2011) 57:2160–8. 10.1016/j.jacc.2010.09.084 21596231 PMC9689753

[34] Gosselin-Badaroudine P Keller DI Huang H Pouliot V Chatelier A Osswald S. A proton leak current through the cardiac sodium channel is linked to mixed arrhythmia and the dilated cardiomyopathy phenotype. PLoS ONE (2012) 7:e38331. 10.1371/journal.pone.0038331 22675453 PMC3365008

[35] Sokolov S Scheuer T Catterall WA. Gating pore current in an inherited ion channelopathy. Nature (2007) 446:76–8. 10.1038/nature05598 17330043

[36] Nguyen TP Wang DW Rhodes TH George ALJr. Divergent biophysical defects caused by mutant sodium channels in dilated cardiomyopathy with arrhythmia. Circ Res. (2008) 102:364–71. 10.1161/CIRCRESAHA.107.164673 18048769

[37] Starace DM Bezanilla F. A proton pore in a potassium channel voltage sensor reveals a focused electric field. Nature (2004) 427:548–53. 10.1038/nature02270 14765197

[38] Watanabe H Yang T Stroud DM Lowe JS Harris L Atack TC. Striking In vivo phenotype of a disease-associated human SCN5A mutation producing minimal changes *in vitro*. Circulation (2011) 124:1001–11. 10.1161/CIRCULATIONAHA.110.987248 21824921 PMC3297976

[39] Starace DM Bezanilla F. Histidine scanning mutagenesis of basic residues of the S4 segment of the shaker k+ channel. J Gen Physiol. (2001) 117:469–90. 10.1085/jgp.117.5.469 11331357 PMC2233663

[40] Tombola F Pathak MM Isacoff EY. Voltage-sensing arginines in a potassium channel permeate and occlude cation-selective pores. Neuron (2005) 45:379–88. 10.1016/j.neuron.2004.12.047 15694325

[41] Sokolov S Scheuer T Catterall WA. Ion permeation through a voltage- sensitive gating pore in brain sodium channels having voltage sensor mutations. Neuron (2005) 47:183–9. 10.1016/j.neuron.2005.06.012 16039561

[42] Banerjee A Mackinnon R. Inferred motions of the S3a helix during voltage-dependent K+ channel gating. J Mol Biol. (2008) 381:569–80. 10.1016/j.jmb.2008.06.010 18632115 PMC2819426

[43] Tao X Lee A Limapichat W Dougherty DA Mackinnon R. A gating charge transfer center in voltage sensors. Science (2010) 328:67–73. 10.1126/science.1185954 20360102 PMC2869078

[44] Delemotte L Tarek M Klein ML Amaral C Treptow W. Intermediate states of the Kv1.2 voltage sensor from atomistic molecular dynamics simulations. Proc Natl Acad Sci USA. (2011) 108:6109–14. 10.1073/pnas.1102724108 21444776 PMC3076833

[45] Amaral C Carnevale V Klein ML Treptow W. Exploring conformational states of the bacterial voltage-gated sodium channel NavAb via molecular dynamics simulations. Proc Natl Acad Sci USA. (2012) 109:21336–41. 10.1073/pnas.1218087109 23150565 PMC3535635

[46] Tarek M Delemotte L. Omega currents in voltage-gated ion channels: what can we learn from uncovering the voltage-sensing mechanism using md simulations? Acc Chem Res. (2013) 46:2755–62. 10.1021/ar300290u 23697886

[47] Pless SA Galpin JD Niciforovic AP Ahern CA. Contributions of counter-charge in a potassium channel voltage-sensor domain. Nat Chem Biol. (2011) 7:617–23. 10.1038/nchembio.622 21785425 PMC4933587

[48] Delemotte L Klein ML Tarek M. Molecular dynamics simulations of voltage-gated cation channels: insights on voltage-sensor domain function and modulation. Front Pharmacol. (2012) 3:97. 10.3389/fphar.2012.00097 22654756 PMC3361024

[49] Jiang D Gamal El-Din TM Ing C Lu P Pomes R Zheng N. Structural basis for gating pore current in periodic paralysis. Nature (2018) 557:590–4. 10.1038/s41586-018-0120-4 29769724 PMC6708612

[50] Gosselin-Badaroudine P Delemotte L Moreau A Klein ML Chahine M. Gating pore currents and the resting state of Nav1.4 voltage sensor domains. Proc Natl Acad Sci USA. (2012) 109:19250–5. 10.1073/pnas.1217990109 23134726 PMC3511134

[51] Moreau A Gosselin-Badaroudine P Chahine M. Biophysics, pathophysiology, and pharmacology of ion channel gating pores. Front Pharmacol. (2014) 5:53. 10.3389/fphar.2014.00053 24772081 PMC3982104

[52] Moreau A Gosselin-Badaroudine P Delemotte L Klein ML Chahine M. Gating pore currents are defects in common with two Nav1.5 mutations in patients with mixed arrhythmias and dilated cardiomyopathy. J Gen Physiol. (2015) 145:93–106. 10.1085/jgp.201411304 25624448 PMC4306709

[53] Moreau A Gosselin-Badaroudine P Boutjdir M Chahine M. Mutations in the voltage sensors of domains I and II of Nav1.5 that are associated with arrhythmias and dilated cardiomyopathy generate gating pore currents. Front Pharmacol. (2015) 6:301. 10.3389/fphar.2015.00301 26733869 PMC4689871

[54] Campos FV Chanda B Roux B Bezanilla F. Two atomic constraints unambiguously position the S4 segment relative to S1 and S2 segments in the closed state of Shaker K channel. Proc Natl Acad Sci USA. (2007) 104:7904–9. 10.1073/pnas.0702638104 17470814 PMC1876545

[55] Struyk AF Cannon SC. A Na+ channel mutation linked to hypokalemic periodic paralysis exposes a proton-selective gating pore. J Gen Physiol. (2007) 130:11–20. 10.1085/jgp.200709755 17591984 PMC2154364

[56] Klassen TL Spencer AN Gallin WJ. A naturally occurring omega current in a Kv3 family potassium channel from a platyhelminth. BMC Neurosci. (2008) 9:52. 10.1186/1471-2202-9-52 18565223 PMC2443804

[57] Struyk AF Cannon SC. Paradoxical depolarization of BA2+- treated muscle exposed to low extracellular K+: insights into resting potential abnormalities in hypokalemic paralysis. Muscle Nerve (2008) 37:326–37. 10.1002/mus.20928 18041053

[58] Struyk AF Markin VS Francis D Cannon SC. Gating pore currents in DIIS4 mutations of NaV1.4 associated with periodic paralysis: saturation of ion flux and implications for disease pathogenesis. J Gen Physiol. (2008) 132:447–64. 10.1085/jgp.200809967 18824591 PMC2553391

[59] Sokolov S Scheuer T Catterall WA. Ion permeation and block of the gating pore in the voltage sensor of NaV1.4 channels with hypokalemic periodic paralysis mutations. J Gen Physiol. (2010) 136:225–36. 10.1085/jgp.201010414 20660662 PMC2912069

[60] Francis DG Rybalchenko V Struyk A Cannon SC Leaky sodium channels from voltage sensor mutations in periodic paralysis, but not paramyotonia. Neurology (2011) 76:1635–41. 10.1212/WNL.0b013e318219fb57 21490317 PMC3100087

[61] Groome JR Lehmann-Horn F Fan C Wolf M Winston V Merlini L. Nav1.4 mutations cause hypokalaemic periodic paralysis by disrupting IIIS4 movement during recovery. Brain (2014) 137:998–1008. 10.1093/brain/awu015 24549961 PMC3959555

[62] Moreau A Gosselin-Badaroudine P Chahine M. Molecular biology and biophysical properties of ion channel gating pores. Q Rev Biophys. (2014) 47:364–88. 10.1017/S0033583514000109 25382261

[63] Delemotte L Treptow W Klein ML Tarek M. Effect of sensor domain mutations on the properties of voltage-gated ion channels: molecular dynamics studies of the potassium channel Kv1.2. Biophys J. (2010) 99:L72–4. 10.1016/j.bpj.2010.08.069 21044565 PMC2966007

[64] Khalili-Araghi F Tajkhorshid E Roux B Schulten K. Molecular dynamics investigation of the omega-current in the Kv1.2 voltage sensor domains. Biophys J. (2012) 102:258–67. 10.1016/j.bpj.2011.10.057 22339862 PMC3260662

[65] Fuster C Perrot J Berthier C Jacquemond V Charnet P Allard B. Na leak with gating pore properties in hypokalemic periodic paralysis V876E mutant muscle Ca channel. J Gen Physiol. (2017) 149:1139–48. 10.1085/jgp.201711834 29114033 PMC5715907

[66] Jurkat-Rott K Lehmann-Horn F. Do hyperpolarization-induced proton currents contribute to the pathogenesis of hypokalemic periodic paralysis, a voltage sensor channelopathy? J Gen Physiol. (2007) 130:1–5. 10.1085/jgp.200709834 17591982 PMC2154370

[67] Jurkat-Rott K Weber MA Fauler M Guo XH Holzherr BD Paczulla A. K+-dependent paradoxical membrane depolarization and Na+ overload, major and reversible contributors to weakness by ion channel leaks. Proc Natl Acad Sci USA. (2009) 106:4036–41. 10.1073/pnas.0811277106 19225109 PMC2644652

[68] Jurkat-Rott K Groome J Lehmann-Horn F. Pathophysiological role of omega pore current in channelopathies. Front Pharmacol. (2012) 3:112. 10.3389/fphar.2012.00112 22701429 PMC3372090

[69] Ruan Y Liu N Priori SG. Sodium channel mutations and arrhythmias. Nat Rev Cardiol. (2009) 6:337–48. 10.1038/nrcardio.2009.44 19377496

[70] Moreau A Krahn AD Gosselin-Badaroudine P Klein GJ Christe G Vincent Y. Sodium overload due to a persistent current that attenuates the arrhythmogenic potential of a novel LQT3 mutation. Front Pharmacol. (2013) 4:126. 10.3389/fphar.2013.00126 24098284 PMC3787509

[71] Olson TM Michels VV Ballew JD Reyna SP Karst ML Herron KJ. Sodium channel mutations and susceptibility to heart failure and atrial fibrillation. JAMA (2005) 293:447–54. 10.1001/jama.293.4.447 15671429 PMC2039897

[72] Olesen MS Yuan L Liang B Holst AG Nielsen N Nielsen JB. High prevalence of long QT syndrome-associated SCN5A variants in patients with early-onset lone atrial fibrillation. Circulation Cardiovascu Genet. (2012) 5:450–9. 10.1161/CIRCGENETICS.111.962597 22685113

[73] Pieske B Houser SR. [Na+]i handling in the failing human heart. Cardiovasc Res. (2003) 57:874–86. 10.1016/S0008-6363(02)00841-6 12650866

[74] Miake J Marban E Nuss HB. Functional role of inward rectifier current in heart probed by Kir2.1 overexpression and dominant-negative suppression. J Clin Invest. (2003) 111:1529–1536. 10.1172/JCI200317959 12750402 PMC155052

[75] Bett GC Kaplan AD Lis A Cimato TR Tzanakakis ES Zhou Q. Electronic “expression” of the inward rectifier in cardiocytes derived from human-induced pluripotent stem cells. Heart Rhythm (2013) 10:1903–10. 10.1016/j.hrthm.2013.09.061 24055949 PMC3851822

[76] Zaza A Rocchetti M Brioschi A Cantadori A Ferroni A. Dynamic Ca2+-induced inward rectification of K+ current during the ventricular action potential. Circ Res. (1998) 82:947–56. 10.1161/01.RES.82.9.947 9598592

[77] Cannon SC. Voltage-sensor mutations in channelopathies of skeletal muscle. J Physiol. (2010) 588:1887–95. 10.1113/jphysiol.2010.186874 20156847 PMC2901977

[78] Tricarico D Camerino DC. Recent advances in the pathogenesis and drug action in periodic paralyses and related channelopathies. Front Pharmacol. (2011) 2:8. 10.3389/fphar.2011.00008 21687503 PMC3108473

[79] Stergiopoulos K Alvarado JL Mastroianni M Ek-Vitorin JF Taffet SM Delmar M. Hetero-domain interactions as a mechanism for the regulation of connexin channels. Circ Res. (1999) 84:1144–55. 10.1161/01.RES.84.10.1144 10347089

[80] Bukauskas FF Bukauskiene A Bennett MV Verselis VK. Gating properties of gap junction channels assembled from connexin43 and connexin43 fused with green fluorescent protein. Biophys J. (2001) 81:137–52. 10.1016/S0006-3495(01)75687-1 11423402 PMC1301499

[81] Duffy HS Ashton AW O'donnell P Coombs W Taffet SM Delmar M. Regulation of connexin43 protein complexes by intracellular acidification. Circ Res. (2004) 94:215–22. 10.1161/01.RES.0000113924.06926.11 14699011

[82] Benson DW Wang DW Dyment M Knilans TK Fish FA Strieper MJ. Congenital sick sinus syndrome caused by recessive mutations in the cardiac sodium channel gene (SCN5A). J Clin Invest. (2003) 112:1019–28. 10.1172/JCI200318062 14523039 PMC198523

[83] Bezzina CR Rook MB Groenewegen WA Herfst LJ Van Der Wal AC Lam J. Compound heterozygosity for mutations (W156X and R225W) in SCN5A associated with severe cardiac conduction disturbances and degenerative changes in the conduction system. Circ Res. (2003) 92:159–68. 10.1161/01.RES.0000052672.97759.36 12574143

[84] Laurent G Saal S Amarouch MY Beziau DM Marsman RF Faivre L. Multifocal ectopic Purkinje-related premature contractions: a new SCN5A-related cardiac channelopathy. J Am Coll Cardiol. (2012) 60:144–56. 10.1016/j.jacc.2012.02.052 22766342

[85] Saegusa N Moorhouse E Vaughan-Jones RD Spitzer KW. Influence of pH on Ca(2)(+) current and its control of electrical and Ca(2)(+) signaling in ventricular myocytes. J Gen Physiol. (2011) 138:537–59. 10.1085/jgp.201110658 22042988 PMC3206307

[86] Thorsen K Dam VS Kjaer-Sorensen K Pedersen LN Skeberdis VA Jurevicius J. Loss-of-activity-mutation in the cardiac chloride-bicarbonate exchanger AE3 causes short QT syndrome. Nat Commun. (2017) 8:1696. 10.1038/s41467-017-01630-0 29167417 PMC5700076

[87] Fabiato A Fabiato F. Effects of pH on the myofilaments and the sarcoplasmic reticulum of skinned cells from cardiace and skeletal muscles. J Physiol. (1978) 276:233–55. 10.1113/jphysiol.1978.sp012231 25957 PMC1282422

[88] Chandra M Montgomery DE Kim JJ Solaro RJ. The N-terminal region of troponin T is essential for the maximal activation of rat cardiac myofilaments. J Mol Cell Cardiol. (1999) 31:867–80. 10.1006/jmcc.1999.0928 10329214

[89] Zhang Z Feng HZ Jin JP. Structure of the NH2-terminal variable region of cardiac troponin T determines its sensitivity to restrictive cleavage in pathophysiological adaptation. Arch Biochem Biophys. (2011) 515:37–45. 10.1016/j.abb.2011.08.013 21924234 PMC3192527

[90] Laitinen-Forsblom PJ Makynen P Makynen H Yli-Mayry S Virtanen V Kontula K. SCN5A mutation associated with cardiac conduction defect and atrial arrhythmias. J Cardiovasc Electrophysiol. (2006) 17:480–5. 10.1111/j.1540-8167.2006.00411.x 16684018

[91] Gui J Wang T Jones RP Trump D Zimmer T Lei M. Multiple loss-of-function mechanisms contribute to SCN5A-related familial sick sinus syndrome. PLoS ONE (2010a) 5:e10985. 10.1371/journal.pone.0010985 20539757 PMC2881866

[92] Gui J Wang T Trump D Zimmer T Lei M. Mutation-specific effects of polymorphism H558R in SCN5A-related sick sinus syndrome. J Cardiovasc Electrophysiol. (2010) 21:564–73. 10.1111/j.1540-8167.2010.01762.x 20384651

[93] Hayano M Makiyama T Kamakura T Watanabe H Sasaki K Funakoshi S. Development of a patient-derived induced pluripotent stem cell model for the Investigation of SCN5A-D1275N-related cardiac sodium channelopathy. Circ J. (2017) 81:1783–91 10.1253/circj.CJ-17-0064 28637969

[94] Moreau A Janin A Millat G Chevalier P. Cardiac voltage-gated sodium channel mutations associated with left atrial dysfunction and stroke in children. EP Europace, euy041-euy041. (2018). 10.1093/europace/euy041 29579189

[95] Huttner IG Trivedi G Jacoby A Mann SA Vandenberg JI Fatkin D. A transgenic zebrafish model of a human cardiac sodium channel mutation exhibits bradycardia, conduction-system abnormalities and early death. J Mol Cell Cardiol. (2013) 61:123–32. 10.1016/j.yjmcc.2013.06.005 23791817

[96] Xiao Y Blumenthal K Cummins TR. Gating-pore currents demonstrate selective and specific modulation of individual sodium channel voltage-sensors by biological toxins. Mol Pharmacol. (2014) 86:159–67. 10.1124/mol.114.092338 24898004 PMC4127926

